# Metabolomic Analysis of Feces vs. Cecum Content in Animals: A Comparative Study Investigated by ^1^H-NMR

**DOI:** 10.3390/metabo15090565

**Published:** 2025-08-22

**Authors:** Xiexin Li, Yang Li, Xin Nie, Chenglin Zhu, Qiqi Luo, Luca Laghi, Gianfranco Picone

**Affiliations:** 1 College of Culinary and Food Science Engineering, Sichuan Tourism University, Chengdu 610100, China; lixiexin@sctu.edu.cn (X.L.); yangli0505@163.com (Y.L.); 2 College of Pharmacy and Food, Southwest Minzu University, Chengdu 610041, China; 3 School of Laboratory Medicine, Chengdu Medical College, Chengdu 610500, China; 4 Department of Agricultural and Food Sciences, University of Bologna, 47521 Cesena, Italy; l.laghi@unibo.it (L.L.); gianfranco.picone@unibo.it (G.P.)

**Keywords:** feces, cecum content, metabolome, ^1^H-NMR, 16S rRNA

## Abstract

**Background:** Feces and cecum content are commonly involved in metabolomic analysis to understand the gut metabolic profile of the host, while, in fact, they are different. Feces represent the terminal excretory product after extensive host enzymatic digestion, absorption, and significant modification by the distal gut microbiota. In contrast, cecum content reflects the localized, in situ metabolic microenvironment at that specific site. However, it is worth noting that feces are the most accessible sample type for non-invasive studies, which could be considered proxies for cecum content in some specific cases. Unfortunately, the validity of fecal samples as an alternative to cecum content has rarely been assessed. **Methods**: The current study attempted to illustrate the distinct metabolomic and microbiota features of feces and cecum content in eight animals (mouse, pig, chicken, duck, rabbit, Gansu yak, Sichuan yak, and sheep) by means of ^1^H-NMR and 16S rRNA, respectively. **Results**: A total of 116 molecules were characterized in feces and cecum content samples. Among them, 22 molecules were shared in all groups. Taking advantage of the univariate analysis, twenty-seven of the quantified molecules were significantly different between feces and cecum content, mainly pertaining to amino acids and organic acids. Moreover, in terms of mammals and non-mammals, short-chain fatty acids could be considered the main factor discriminating the metabolomic profiles between feces and cecum content. Furthermore, to better understand the mechanism of their metabolomic differences, 16S rRNA sequencing analysis was performed on feces and cecum content samples of mice, which is the most widely used animal model. The result showed that the Ace, Shannon, and Sobs indexes in feces were significantly higher than those of cecum content (*p* < 0.05). At the phylum and genus levels, the microbiota structures of feces and cecum content were similar, while the relative abundances of their microbiota exhibited distinct features. **Conclusions**: The present study could reduce this gap in information by characterizing, for the first time, the metabolomic differences between feces and cecum content using ^1^H-NMR. Moreover, this study is meant as a reference guide for researchers wishing to apply a metabolomics approach to the gut of the host.

## 1. Introduction

Metabolomics is widely utilized to unveil the metabolic characteristics of biofluids in distinct physiological states, thereby shedding light on their physiological and pathological processes. In the realm of animal research, the application of metabolomics has yielded noteworthy outcomes [1,2,3]. Feces and cecum content serve as pivotal indicators of gut microbial metabolism, and their metabolomic profiles are influenced by various factors, primarily pertaining to physiological states [4]. For instance, our previous study indicated that the concentrations of sixteen molecules in feces were found to be significantly different between mastitic and healthy cows [5]. Another previous study showed that a high-fat diet could significantly elevate the concentrations of acetone and methionine while decreasing the levels of methanol, arabinose, acetate, and 3-hydroxyphenylacetate in the cecum contents of mice [6].

Fecal metabolomics is increasingly employed to examine the composition and dynamic patterns of gut microbial metabolism in animal models by taking advantage of its simple and non-invasive features. Fecal sample collection is not harmful to animals, so it is suitable for monitoring metabolomic changes in the host in a longitudinal study [7]. Zhang et al. found that major changes in fecal metabolites were confirmed for HFID-fed mice, including those related to entero-hepatic circulation (i.e., bile acids), tryptophan metabolism (e.g., indole derivatives), and lipid metabolism (e.g., lipoic acid), as well as increased antioxidants, including isorhapontigenin [8]. Kang et al. found that the contents of malic acid, malonic acid, succinic acid, and fumaric acid in the normal mice’s feces were 2–10 times higher than those of obese mice [9]. In addition, gut content, being a biological sample in contact with the gut microbiota, offers a direct insight into the gut microbiota metabolism [10,11,12,13]. Zhou et al. found that 0.4% of *Ampelopsis grossedentata* extract supplementation significantly downregulated the levels of *p*-cresol sulfate and cholesterol sulfate in broilers’ gut content, through a UPLC-MS-based metabolomic approach [14].

However, the metabolomic point of view obtained from feces and cecum content is distinct in several cases. Therefore, the proper selection of feces and cecum contents for metabolomic analyses within the same animal model under identical treatments is still a question mark. For example, Van Hul et al. attempted to compare the effects of soluble corn fiber and fructooligosaccharides on the metabolism of high-fat-diet-fed mice. The results showed that the levels of isobutyric acid, isovaleric acid, and valeric acid in cecum content were significantly different among the groups investigated. In parallel, the same molecules were found to be non-significantly different in feces, even if other short-chain fatty acids, like acetic acid and butyric acid, were [15].

Currently, there is a prevalence of metabolomics analyses focusing on either cecum contents or feces in animal models. However, representative studies comparing the similarities and differences of metabolomic profiles between feces and cecum content are relatively limited. In order to fill such gaps, the present comparative study attempted to investigate by ^1^H-NMR the metabolomic differences between feces and cecum content in several commonly observed animals, namely mouse, pig, chicken, duck, rabbit, Gansu yak, Sichuan yak, and sheep. The findings of this study will provide a reference guide for researchers seeking to apply a metabolomics approach to the feces and cecum content and provide new lines of evidence for researchers to select the best-suited samples for their investigations.

## 2. Materials and Methods

### 2.1. Sampling

The experimental designs and protocols of the current study received approval from the Southwest Minzu University Animal Ethics Committee (Protocol NO. SWUN-A-0060) and adhered to the recommendations outlined in the academy’s guidelines for animal research.

All feces and cecum content samples, involving pig, chicken, duck, rabbit, Gansu yak, Sichuan yak, and sheep, were randomly collected from a slaughterhouse in Chengdu, Sichuan. Mice were housed in the laboratory for one week. Then, feces and cecum content samples were collected right after sacrifice. A total of 80 samples, belonging to eight animals, were considered for the present study (5 feces and 5 cecum content samples per animal). All samples were promptly transported to the laboratory with ice and stored at −80 °C for the subsequent analysis.

### 2.2. Metabolomic Analysis

**Figure 1:**
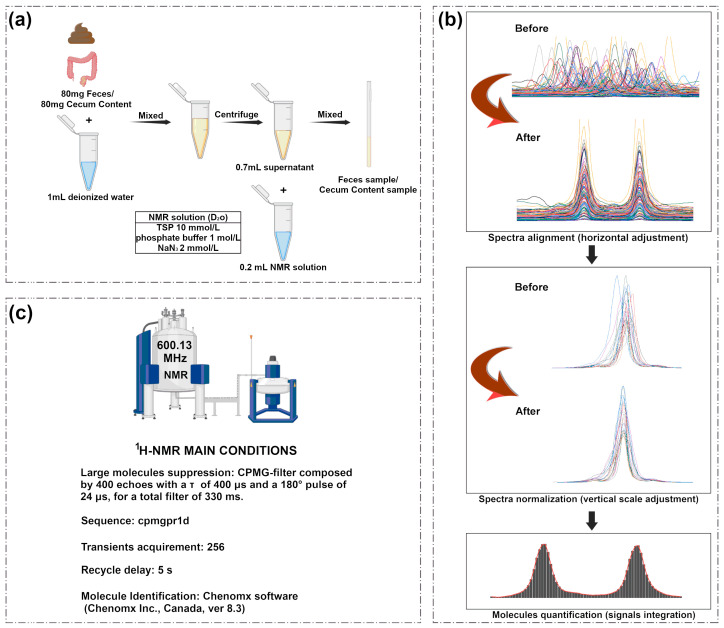
(**a**) Sample preparation of feces and cecum contents; (**b**) applied to the entire spectral array using probabilistic quotient normalization; (**c**) ^1^H-NMR main conditions.

NMR solutions were prepared in accordance with previous works of some of the authors [16], as indicated in Figure 1.

As shown in Figure 1a, we added 80 mg of each stool/cecum content sample to 1 mL of deionized water in an Eppendorf tube and vortexed for 5 min, followed by centrifugation for 15 min at 18,630× *g* and 4 °C. Subsequently, we moved 0.7 mL of supernatant to a new Eppendorf tube, together with 0.2 mL of NMR analysis solution described above. After a final centrifugation, the supernatant was transferred to an NMR tube. As previously reported [17], the ^1^H-NMR spectra were recorded at 298 K using an AVANCE III spectrometer (Bruker, Wuhan, China) at 600.13 MHz.

As shown in Figure 1b, the baseline ^1^H-NMR spectrum was adjusted by peak detection according to the “rolling ball” principle [18] implemented in the baseline R package 4.4.1 [19]. Differences in water and fiber content among samples were taken into consideration by probabilistic quotient normalization [20] applied to the entire spectra array.

As shown in Figure 1c, the broad resonance signal from the macromolecule was suppressed by a CPMG filter consisting of 400 echoes with 400 μs of τ, 24 μs of 180° pulses, and 330 ms of total filter. The HOD residual signal was suppressed by a prediction method. This was accomplished by using cpmgpr1d sequences from a library of standard pulse sequences. Each spectrum was acquired to summarize 256 transients using 32 K data points in a 7184 Hz spectral window with an acquisition time of 2.28 s. The acquisition time was 2.28 s.

In order to apply NMR as a quantitative technique [21], the cycle delay was set to 5 s, keeping in mind the relaxation time of the proton under investigation. These signals were assigned by calculating their chemical shifts and diversity using the Chenomx software library (Chenomx Incorporation, Edmonton, AL, Canada, version 8.4). Molecule quantification was carried out by means of signal integration.

### 2.3. Microbiome Analysis

**Figure 2:**
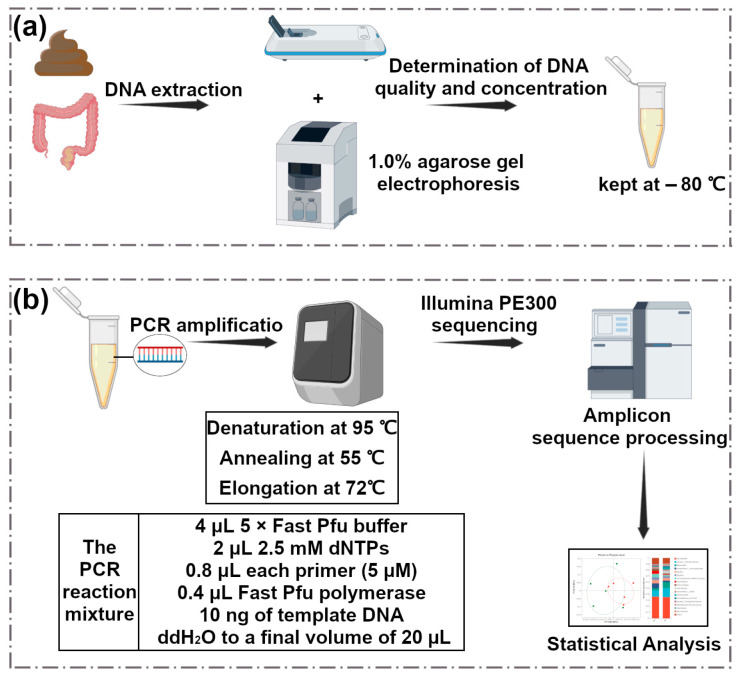
Pictorial representation of (**a**) DNA extraction, (**b**) PCR amplification, Illumina PE300 sequencing, and amplicon sequence processing and analysis.

Total genomic DNA of the microbial community was extracted from feces and cecum contents according to the instructions of the E.Z.N.A.^®^ soil DNA kit (Omega Bio-tek, Norcross, GA, USA). As shown in Figure 2, the quality of the DNA was checked by agarose gel electrophoresis, and the concentration and purity of DNA were determined by NanoDrop2000 (Thermo Fisher Scientific, Waltham, MA, USA). The hypervariable region V3-V4 of the bacterial 16S rRNA gene was amplified with primer pairs 338F (5′-ACTCCTACGGGAGGCAGCAG-3′) and 806R (5′-GGACTACHVGGGTWTCTAAT-3′) [22] by T100 Thermal Cycler PCR thermocycler (BIO-RAD, Hercules, CA, USA).

PCR amplification was performed, and the recovered products were detected and quantified using Qubit 4.0 (Thermo Fisher Scientific, USA). The purified PCR products were constructed using a library with the NEXTFLEX^®^ Rapid DNA-Seq Kit. High-throughput sequencing data were analyzed for species taxonomy of ASVs based on the Silva 16S rRNA gene database (v 138) using the Naive Bayes (or Vsearch 2.30.0, or Blast) classifier in Qiime2 [23], and 16S function prediction analysis was performed using PICRUSt2 [24] (version 2.2.0) software.

### 2.4. Statistical Analysis

Statistical analysis on metabolomic data was conducted in the R computational language [25]. Molecules whose concentration varied between groups were assessed using a *t*-test. For this purpose, a cut-off *p*-value of 0.05 was accepted. Prior to the *t*-test, variables that were not normally distributed were normally distributed by following Box and Cox [26]. To highlight the trends underlying the structure of the samples, we relied on PLS-DA models on the molecules accepted by the above-described univariate analysis [27]. Microbiota data analysis was performed on the Meggie BioCloud platform (https://cloud.majorbio.com, accessed on 15 December 2024) [28]. A clustering correlation heatmap was generated using the OmicStudio tools (https://www.omicstudio.cn, accessed on 15 December 2024) [29].

## 3. Results

### 3.1. Metabolomic Characterizations of Feces and Cecum Content

**Figure 3:**
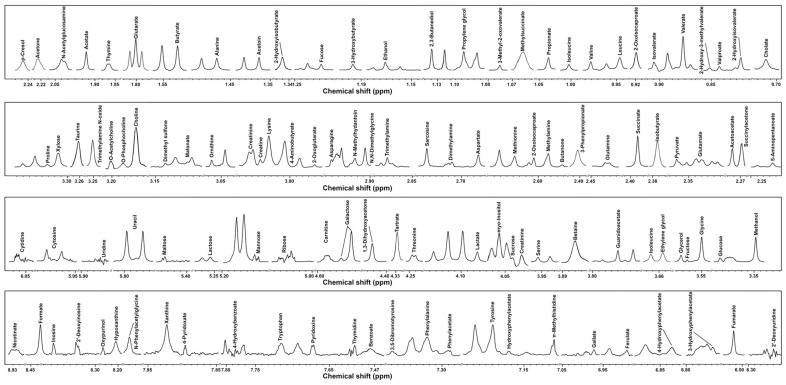
A representative ^1^H-NMR spectrum of feces and cecum content from all samples.

Taking advantage of ^1^H-NMR, a total of 116 molecules were identified and quantified in feces and cecum content samples from all the involved animals. Information on the above molecules is provided in Supplementary Materials Table S1. A representative spectrum, which contains all the molecules identified from all samples, is shown in Figure 3. As the molecules characterized from feces and cecum content of the same animal are identical, in order to visually identify the metabolomic differences among distinct animals, an upset plot was generated, as shown in Supplementary Materials Figure S1.

### 3.2. Comparative Metabolomic Analysis of Feces and Cecum Content in Mammals and Non-Mammals

**Figure 4:**
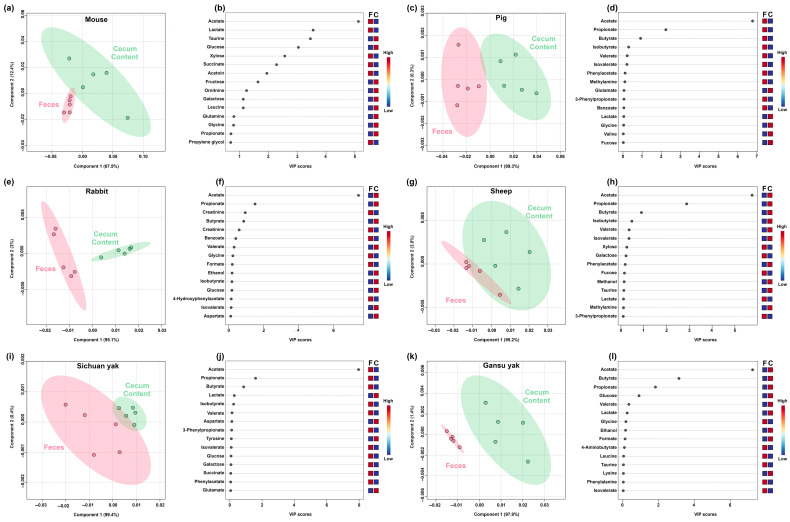
PLS-DA model built on the basis of molecular concentrations characterized by ^1^H-NMR for each mammal model. (**a**,**b**) Mouse; (**c**,**d**) Pig; (**e**,**f**) Rabbit; (**g**,**h**) Sheep; (**i**,**j**) Sichuan yak; (**k**,**l**) Gansu yak.

As shown in Figure 4, PLS-DA analysis indicated that the main differences between feces and cecum content regarded short-chain fatty acids (SCFAs), namely acetate, butyrate, and propionate. However, it is worth noting that the trend of SCFAs in each mammal model was peculiar. In detail, mouse, pig, and Sichuan yak had higher levels of SCFAs in feces, while rabbit, sheep, and Gansu yak showed an opposite trend.

**Figure 5:**
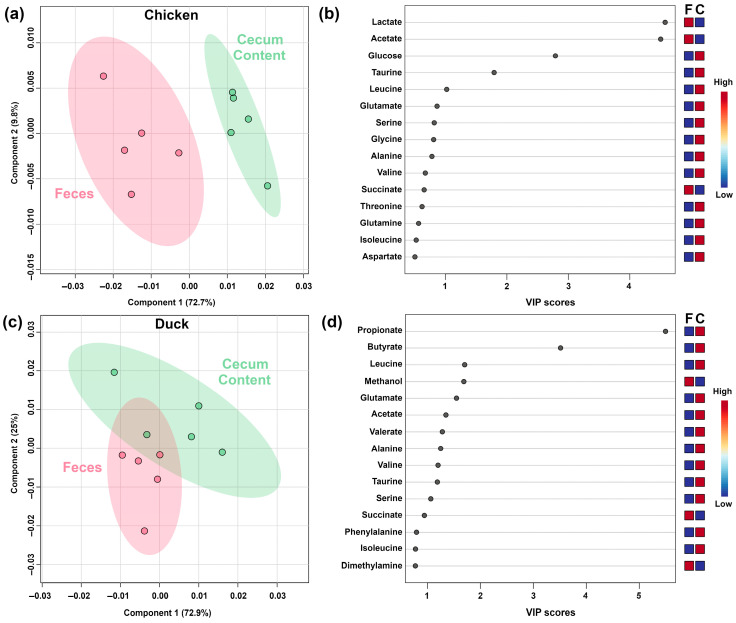
PLS-DA model built on the concentrations of molecules characterized by ^1^H-NMR in each non-mammal. (**a**,**b**) Chicken; (**c**,**d**) Duck.

Figure 5 shows PLS-DA models built on feces and cecum content of non-mammals. It is remarkable to notice that, similarly to the mammal models studied, the main differences between feces and cecum content were again SCFAs, with opposite overall trends between chicken and duck.

### 3.3. Microbiome Characterizations of Feces and Cecum Content in Mouse

In order to underline the biological mechanisms leading to the metabolomic differences observed between feces and cecum content, the samples from the mouse, which is the most commonly used animal model in experiments, were subjected to 16S rRNA analysis. Furthermore, correlations between metabolomic and microbiome profiles of feces and cecum content were investigated.

**Figure 6:**
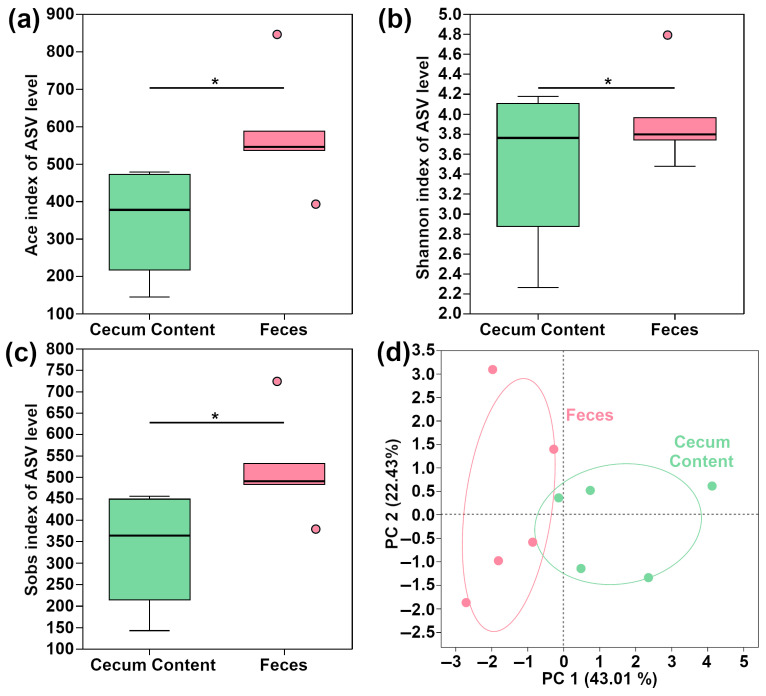
α-Diversity and *β*-diversity of feces and cecum content: Ace index (**a**), Shannon index (**b**), Sobs index (**c**), and PCA plot of *β*-diversity (**d**); * denotes significant differences between the two groups (*p* < 0.05).

As shown in Figure 6, the Ace, Shannon, and Sobs indexes in feces were significantly higher than those of cecum content (*p* < 0.05). In addition, the PCA plot of *β*-diversity double confirmed the differences of microbiome features between feces and cecum content.

**Figure 7:**
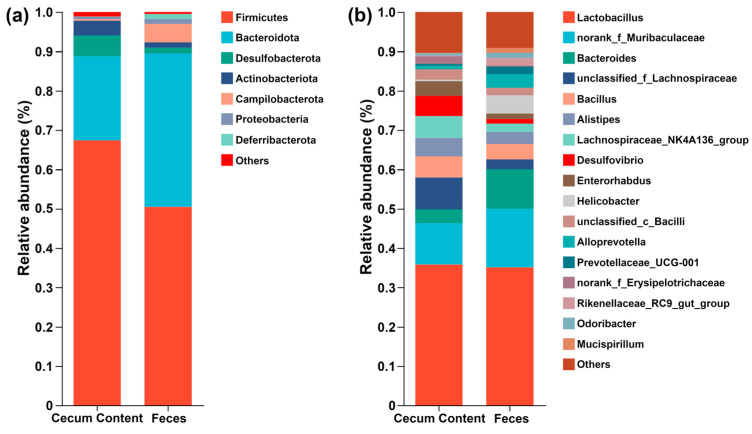
The composition of different microorganisms between feces and cecum content samples at the phylum level (**a**) and the genus level (**b**) (n = 5).

In order to address the difference in microorganisms’ composition between feces and cecum content, a stacking diagram was created at the phylum and genus levels, as shown in Figure 7. At the phylum level, *Firmicutes* and *Bacteroidota* were the dominant microorganisms, followed by *Desulfobacterota* and *Campilobacterota* in cecum content and feces, respectively. At the genus level, *Lactobacillus* and *norank_f_Muribaculaceae* could be considered as the dominant microorganisms, followed by others and *Bacteroides* in cecum content and feces, respectively.

**Figure 8:**
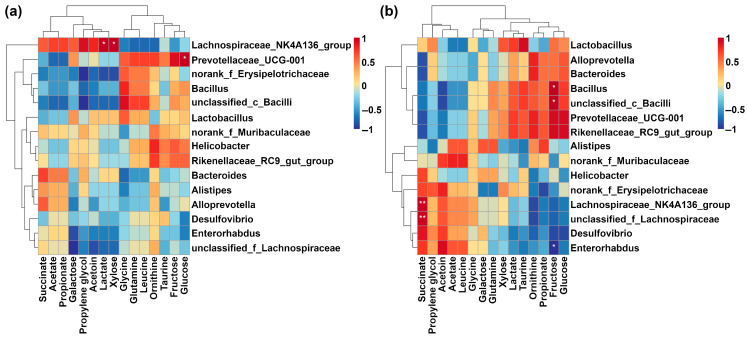
Heatmap of Spearman correlation between VIP molecules and the TOP 15 microorganisms at the genus level: (**a**) cecum content; (**b**) feces. * *p* < 0.05, ** *p* < 0.01.

As shown in Figure 8a, the level of glucose was positively correlated with the abundance of *Prevotellaceae_UCG-001*. Moreover, the amounts of lactate and xylose were positively correlated with the abundance of *Lachnospiraceae_NK4A136_group*. Referring to feces, the concentration of fructose was positively related to the abundance of *Bacillus* and *unclassified_c_Bacilli*, while negatively related to the amount of *Enterorhabdus*. In addition, the concentration of succinate was positively correlated with the amounts of *Lachnospiraceae_NK4A136_group* and *unclassified_f_Lachnospiraceae*, as shown in Figure 8b.

## 4. Discussion

In recent years, numerous studies have been conducted to analyze the metabolome of biofluids in animal models, attempting, for example, to investigate the features connected to health and disease [30]. Fecal metabolomics is widely utilized to provide insights into gut–microbial co-metabolism, taking advantage of its non-invasiveness and ease of sample collection. In parallel, cecum content can allow for a more direct observation of the connections between gut flora and host metabolism, which counterbalances the complex sample collection procedures [31]. However, it is noteworthy that the trends highlighted in feces and cecum content have been found to be different in some cases. For example, in a metabolomics study on cecum content, Zhou et al. found that green tea catechin epigallocatechin gallate (EGCG) could alleviate high-fat-diet-induced obesity in mice, by increasing the gut microbial abundance and reducing short-chain fatty acids (SCFAs) [32]. However, another metabolomics study on the feces of the same animal found that administration of tea water extracts could improve glucose tolerance and significantly increase the production of SCFAs [33]. Therefore, investigating the metabolomic differences between feces and cecum content appears of the utmost importance. On one hand, these studies can assist a researcher in choosing the best samples for his/her experiments. On the other hand, he/she can understand whether a certain feature is connected to the performed experiment or the selected matrix.

The number of molecules quantified in the present study is higher than in previous studies based on the same technique and involving the same animals and biofluids. Examples can be found for mouse feces (89 vs. 67) [34]; mouse cecum content (89 vs. 33) [35]; pig feces (52 vs. 39) [36]; rabbit cecum content (59 vs. 29) [37]; and sheep feces (43 vs. 28) [38].

In mammals, dietary carbohydrates are mainly fermented into SCFAs, such as acetate, propionate, and butyrate. As one of the most abundant SCFAs, acetate could be formed from pyruvate by acetogenic bacteria [39]. Propionate was reported to be mainly produced from succinate by *Bacteroidetes* [40]. In meat rabbits, Fang et al. found that fecal SCFAs levels were potentially correlated with high and low fattening body weights. In detail, the level of butyrate in feces was significantly positively related to finishing weight, while the concentrations of acetate and propionate showed a positive and negative association with finishing weight, respectively [41]. As suggested by Zhao et al., propionate and butyrate are mainly produced in the hindgut of pigs [42]. Butyrate plays a crucial role in intestinal homeostasis as a major energy source for colonocytes, and it is important for maintaining tissue barrier function [43]. Moreover, large quantities of acetate and lactate could facilitate the synthesis of butyrate in the gut by butyrate-producing bacteria [44,45]. Xylooligosaccharide (XOS) administration could significantly increase the levels of SCFAs, both in feces and cecum content, of high-fat-diet-induced obese mice, which could be linked to its modulation by gut microorganisms like *Prevotella* and *Paraprevotella* [46,47]. In sheep, the trends of SCFAs have been found to differ partially between feces and cecum content under N-carbamoylglutamate supplementation. In detail, Zhang et al. found that N-carbamoylglutamate supplementation elevated the levels of acetate, butyrate, and propionate in the colon of lambs [48], while it reduced the level of acetate in feces, as assessed by Ma et al. [49].

In the gut, the host-derived and gut microbiota-derived proteases and peptidases break down dietary protein to amino acids, which are absorbed by the host or reach the colon, where they are further metabolized to SCFAs, including acetate, butyrate, propionate, and BCFAs by colonic microbes [50,51]. In the present study, compared to feces, higher levels of amino acids in cecum content could be linked to their reabsorption and metabolism in the colon. Pyruvate is a crucial intermediate for propionate production through gluconeogenesis and glycolysis [52,53]. Xylose is mainly absorbed in the small intestine at a rate slower than that of galactose and glucose [54,55], which could promote the growth and reproduction of anaerobic bacteria, such as *Bacteroidota* [56].

In order to further underline the reasons for the metabolomic differences we found between cecum content and feces, as the most commonly used animal model, mouse feces and cecum content were collected specifically to obtain their microbiome’s features by means of 16S rRNA analysis. At the phylum level, we found that *Firmicutes* and *Bacteroidota* were the dominant microorganisms in feces and cecum contents, which could be attributed to the digestion of dietary fiber and polysaccharides [57,58]. The distinct ratio between *Firmicutes* and *Bacteroidota* could result in the different concentrations of SCFAs between feces and cecum content [59]. *Unclassified_f_Lachnospiraceae* and *Lachnospiracae_NK4A136_group* were shown to contribute to the production of butyrate [60,61]. *Bacillus* has the ability to digest fructose and is of interest as a potential probiotic for the treatment of metabolic disorders [62,63,64], which has been highlighted by correlation analysis in the present study. Similar to *Bacillus*, *Prevotellaceae_UCG-001* is a bacterium that has the ability to produce SCFAs. Decreased quantities of *Prevotellaceae_UCG-001* in feces and cecum content have been confirmed to be related to several diseases, such as ulcerative colitis [65], chemical poisoning [66], and inflammatory bowel disease [67].

## 5. Conclusions

The objective of the present study was to investigate the metabolomic differences between feces and cecum content using ^1^H-NMR. A total of 116 molecules were characterized in feces and cecum content samples of various animals, a number higher than any previous study based on ^1^H-NMR. Taking advantage of uni- and multivariate analyses, twenty-seven of the quantified molecules were found significantly different between feces and cecum content, mainly pertaining to amino acids and organic acids groups. Moreover, both in mammals and non-mammals, SCFAs could be considered the main factor discriminating the metabolomic profiles of feces and cecum content, probably in connection with the action of the microbiome. It is noteworthy that this study has several limitations for future investigation. As samples were collected randomly from a slaughterhouse, future research should account for influential factors, such as dietary regimens and feeding environments. Additionally, while only murine microbiota was analyzed, incorporating microbiota data from other animal species would strengthen the reference value of findings. Finally, future studies should evaluate interspecies variation in both metabolomic profiles and microbiota characteristics across animals.

## Data Availability

Data from this study are available upon request.

## Supplementary Materials

The following supporting information can be downloaded at: https://www.mdpi.com/article/10.3390/metabo15090565/s1, Figure S1: Upset plot showing metabolites in common and specific to the eight animals. Table S1: Information of molecules characterized by 1H-NMR.

## References

[1] Su T., Tan Y., Tsui M.S., Yi H., Fu X.Q., Li T., Chan C.L., Guo H., Li Y.X., Zhu P.L. (2016). Metabolomics Reveals the Mechanisms for the Cardiotoxicity of Pinelliae Rhizoma and the Toxicity-Reducing Effect of Processing. Sci. Rep..

[2] Shi H., Hu L., Chen S., Bao W., Yang S., Zhao X., Sun C. (2017). Metabolomics Analysis of Urine from Rats Administered with Long-Term, Low-Dose Acrylamide by Ultra-Performance Liquid Chromatography-Mass Spectrometry. Xenobiotica.

[3] Jin X., Yang H., Coldea T.E., Xu Y., Zhao H. (2021). Metabonomic Analysis Reveals Enhanced Growth and Ethanol Production of Brewer’s Yeast by Wheat Gluten Hydrolysates and Potassium Supplementation. LWT.

[4] Nelson A.D., Camilleri M., Chirapongsathorn S., Vijayvargiya P., Valentin N., Shin A., Erwin P.J., Wang Z., Hassan Murad M. (2017). Comparison of Efficacy of Pharmacological Treatments for Chronic Idiopathic Constipation: A Systematic Review and Network Meta-Analysis. Gut.

[5] Zhu C., Zhang Q., Zhao X., Yang Z., Yang F., Yang Y., Tang J., Laghi L. (2023). Metabolomic Analysis of Multiple Biological Specimens (Feces, Serum, and Urine) by 1H-NMR Spectroscopy from Dairy Cows with Clinical Mastitis. Animals.

[6] Zhang Q., Cheng J., Jiang X., Tang J., Zhu C., Chen H., Laghi L. (2023). Metabolomic Characteristics of Cecum Contents in High-Fat-Diet-Induced Obese Mice Intervened with Different Fibers. Foods.

[7] Stanley D., Geier M.S., Chen H., Hughes R.J., Moore R.J. (2015). Comparison of Fecal and Cecal Microbiotas Reveals Qualitative Similarities but Quantitative Differences. BMC Microbiol..

[8] Zhang Z., Chen X., Cui B. (2021). Modulation of the Fecal Microbiome and Metabolome by Resistant Dextrin Ameliorates Hepatic Steatosis and Mitochondrial Abnormalities in Mice. Food Funct..

[9] Kang Y., Zhang B., Li H., Huang G., Lv H., Jiang K. (2023). Differentiation of Obese and Healthy Mice by Analyzing the Carboxylic Acids in the TCA Cycle in Their Feces: Determination of Chelating Carboxylic Acids in Feces. Talanta Open.

[10] Chen Y., Dinges M.M., Green A., Cramer S.E., Larive C.K., Lytle C. (2020). Absorptive Transport of Amino Acids by the Rat Colon. Am. J. Physiol. Gastrointest Liver Physiol..

[11] Heinzmann S.S., Schmitt-Kopplin P. (2015). Deep Metabotyping of the Murine Gastrointestinal Tract for the Visualization of Digestion and Microbial Metabolism. J. Proteome Res..

[12] Tian Y., Zhang L., Wang Y., Tang H. (2012). Age-Related Topographical Metabolic Signatures for the Rat Gastrointestinal Contents. J. Proteome Res..

[13] Dougal K., Harris P.A., Edwards A., Pachebat J.A., Blackmore T.M., Worgan H.J., Newbold C.J. (2012). A Comparison of the Microbiome and the Metabolome of Different Regions of the Equine Hindgut. FEMS Microbiol. Ecol..

[14] Zhou L., Li H., Hou G., Wang J., Zhou H., Wang D. (2022). Effects of Vine Tea Extract on Meat Quality, Gut Microbiota and Metabolome of Wenchang Broiler. Animals.

[15] Van Hul M., Karnik K., Canene-Adams K., De Souza M., Van den Abbeele P., Marzorati M., Delzenne N.M., Everard A., Cani P.D. (2020). Comparison of the Effects of Soluble Corn Fiber and Fructooligosaccharides on Metabolism, Inflammation, and Gut Microbiome of High-Fat Diet-Fed Mice. Am. J. Physiol.-Endocrinol. Metab..

[16] Zhu C., Jin L., Luo B., Zhou Q., Dong L., Li X., Zhang H., Huang Y., Li C., Zou L. (2022). Dominant Components of the Giant Panda Seminal Plasma Metabolome, Characterized By1 H-NMR Spectroscopy. Animals.

[17] Zhu C., Petracci M., Li C., Fiore E., Laghi L. (2020). An Untargeted Metabolomics Investigation of Jiulong Yak (Bos Grunniens) Meat by 1H-NMR. Foods.

[18] Kneen M.A., Annegarn H.J. (1996). Algorithm for Fitting XRF, SEM and PIXE X-Ray Spectra Backgrounds. Nucl. Instrum. Methods Phys. Res. B..

[19] Liland K.H., Almøy T., Mevik B.H. (2010). Optimal Choice of Baseline Correction for Multivariate Calibration of Spectra. Appl. Spectrosc..

[20] Dieterle F., Ross A., Schlotterbeck G., Senn H. (2006). Probabilistic Quotient Normalization as Robust Method to Account for Dilution of Complex Biological Mixtures. Application In1H NMR Metabonomics. Anal. Chem..

[21] Laghi L., Zhu C., Campagna G., Rossi G., Bazzano M., Laus F. (2018). Probiotic Supplementation in Trained Trotter Horses: Effect on Blood Clinical Pathology Data and Urine Metabolomic Assessed in Field. J. Appl. Physiol..

[22] Liu C., Zhao D., Ma W., Guo Y., Wang A., Wang Q., Lee D.J. (2016). Denitrifying Sulfide Removal Process on High-Salinity Wastewaters in the Presence of *Halomonas* sp.. Appl. Microbiol. Biotechnol..

[23] Bolyen E., Rideout J.R., Dillon M.R., Bokulich N.A., Abnet C.C., Al-Ghalith G.A., Alexander H., Alm E.J., Arumugam M., Asnicar F. (2019). Reproducible, Interactive, Scalable and Extensible Microbiome Data Science Using QIIME 2. Nat. Biotechnol..

[24] Douglas G.M., Maffei V.J., Zaneveld J.R., Yurgel S.N., Brown J.R., Taylor C.M., Huttenhower C., Langille M.G.I. (2020). PICRUSt2 for Prediction of Metagenome Functions. Nat. Biotechnol..

[25] R Development Core Team (2011). R: A Language and Environment for Statistical Computing.

[26] Box G.E.P., Cox D.R. (1964). An Analysis of Transformations. J. R. Stat. Soc. Ser. B. (Methodol.).

[27] Hubert M., Rousseeuw P.J., Vanden Branden K. (2005). ROBPCA: A New Approach to Robust Principal Component Analysis. Technometrics.

[28] Schloss P.D., Westcott S.L., Ryabin T., Hall J.R., Hartmann M., Hollister E.B., Lesniewski R.A., Oakley B.B., Parks D.H., Robinson C.J. (2009). Introducing Mothur: Open-Source, Platform-Independent, Community-Supported Software for Describing and Comparing Microbial Communities. Appl. Environ. Microbiol..

[29] Lyu F., Han F., Ge C., Mao W., Chen L., Hu H., Chen G., Lang Q., Fang C. (2023). OmicStudio: A Composable Bioinformatics Cloud Platform with Real-Time Feedback That Can Generate High-Quality Graphs for Publication. iMeta.

[30] Chu X., Jaeger M., Beumer J., Bakker O.B., Aguirre-Gamboa R., Oosting M., Smeekens S.P., Moorlag S., Mourits V.P., Koeken V.A.C.M. (2021). Integration of Metabolomics, Genomics, and Immune Phenotypes Reveals the Causal Roles of Metabolites in Disease. Genome Biol..

[31] Li Z., He H., Ni M., Wang Z., Guo C., Niu Y., Xing S., Song M., Wang Y., Jiang Y. (2022). Microbiome-Metabolome Analysis of the Immune Microenvironment of the Cecal Contents, Soft Feces, and Hard Feces of Hyplus Rabbits. Oxid. Med. Cell Longev..

[32] Zhou J., Ding L., Chen W., Wang Y. (2023). Green Tea Catechin Epigallocatechin Gallate Alleviates High-Fat Diet-Induced Obesity in Mice by Regulating the Gut–Brain Axis. Food Front..

[33] Liu J., Hao W., He Z., Kwek E., Zhao Y., Zhu H., Liang N., Ma K.Y., Lei L., He W.S. (2019). Beneficial Effects of Tea Water Extracts on the Body Weight and Gut Microbiota in C57BL/6J Mice Fed with a High-Fat Diet. Food Funct..

[34] Parker A., Romano S., Ansorge R., Aboelnour A., Le Gall G., Savva G.M., Pontifex M.G., Telatin A., Baker D., Jones E. (2022). Fecal Microbiota Transfer between Young and Aged Mice Reverses Hallmarks of the Aging Gut, Eye, and Brain. Microbiome.

[35] Meng Z., Huang S., Sun W., Yan S., Chen X., Diao J., Zhou Z., Zhu W. (2021). A Typical Fungicide and Its Main Metabolite Promote Liver Damage in Mice through Impacting Gut Microbiota and Intestinal Barrier Function. J. Agric. Food Chem..

[36] Beaumont M., Cauquil L., Bertide A., Ahn I., Barilly C., Gil L., Canlet C., Zemb O., Pascal G., Samson A. (2021). Gut Microbiota-Derived Metabolite Signature in Suckling and Weaned Piglets. J. Proteome Res..

[37] Beaumont M., Paës C., Mussard E., Knudsen C., Cauquil L., Aymard P., Barilly C., Gabinaud B., Zemb O., Fourre S. (2020). Gut Microbiota Derived Metabolites Contribute to Intestinal Barrier Maturation at the Suckling-to-Weaning Transition. Gut Microbes.

[38] Martias C., Gatien J., Roch L., Baroukh N., Mavel S., Lefèvre A., Montigny F., Schibler L., Emond P., Nadal-Desbarats L. (2021). Analytical Methodology for a Metabolome Atlas of Goat’s Plasma, Milk and Feces Using1h-Nmr and Uhplc-Hrms. Metabolites.

[39] Blanco-Pérez F., Steigerwald H., Schülke S., Vieths S., Toda M., Scheurer S. (2021). The Dietary Fiber Pectin: Health Benefits and Potential for the Treatment of Allergies by Modulation of Gut Microbiota. Curr. Allergy Asthma Rep..

[40] Reichardt N., Duncan S.H., Young P., Belenguer A., McWilliam Leitch C., Scott K.P., Flint H.J., Louis P. (2014). Phylogenetic Distribution of Three Pathways for Propionate Production within the Human Gut Microbiota. ISME J..

[41] Fang S., Chen X., Ye X., Zhou L., Xue S., Gan Q. (2020). Effects of Gut Microbiome and Short-Chain Fatty Acids (SCFAs) on Finishing Weight of Meat Rabbits. Front. Microbiol..

[42] Zhao J., Bai Y., Tao S., Zhang G., Wang J., Liu L., Zhang S. (2019). Fiber-Rich Foods Affected Gut Bacterial Community and Short-Chain Fatty Acids Production in Pig Model. J. Funct. Foods.

[43] Kelly C.J., Taylor T., Colgan Correspondence S.P. (2015). Crosstalk between Microbiota-Derived Short-Chain Fatty Acids and Intestinal Epithelial HIF Augments Tissue Barrier Function. Cell Host Microbe.

[44] Louis P., Flint H.J. (2009). Diversity, Metabolism and Microbial Ecology of Butyrate-Producing Bacteria from the Human Large Intestine. FEMS Microbiol. Lett..

[45] Miranda P.M., De Palma G., Serkis V., Lu J., Louis-Auguste M.P., McCarville J.L., Verdu E.F., Collins S.M., Bercik P. (2018). High Salt Diet Exacerbates Colitis in Mice by Decreasing Lactobacillus Levels and Butyrate Production. Microbiome.

[46] Fei Y., Wang Y., Pang Y., Wang W., Zhu D., Xie M., Lan S., Wang Z. (2020). Xylooligosaccharide Modulates Gut Microbiota and Alleviates Colonic Inflammation Caused by High Fat Diet Induced Obesity. Front. Physiol..

[47] Berger K., Burleigh S., Lindahl M., Bhattacharya A., Patil P., Stålbrand H., Nordberg Karlsson E., Hållenius F., Nyman M., Adlercreutz P. (2021). Xylooligosaccharides Increase Bifidobacteria and Lachnospiraceae in Mice on a High-Fat Diet, with a Concomitant Increase in Short-Chain Fatty Acids, Especially Butyric Acid. J. Agric. Food Chem..

[48] Zhang H., Zheng Y., Zha X., Ma Y., Liu X., Elsabagh M., Wang H., Wang M. (2022). Dietary L-Arginine or N-Carbamylglutamate Alleviates Colonic Barrier Injury, Oxidative Stress, and Inflammation by Modulation of Intestinal Microbiota in Intrauterine Growth-Retarded Suckling Lambs. Antioxidants.

[49] Ma W., Yuan M., Chang S., Wang C. (2023). N-Carbamylglutamate Supplementation Regulates Hindgut Microbiota Composition and Short-Chain Fatty Acid Contents in Charollais and Small Tail Han Crossbred Sheep. Front. Vet. Sci..

[50] Ahn I.S., Yoon J., Diamante G., Cohn P., Jang C., Yang X. (2021). Disparate Metabolomic Responses to Fructose Consumption between Different Mouse Strains and the Role of Gut Microbiota. Metabolites.

[51] Neis E.P.J.G., Dejong C.H.C., Rensen S.S. (2015). The Role of Microbial Amino Acid Metabolism in Host Metabolism. Nutrients.

[52] Denton R.M., Halestrap A.P. (1979). Regulation of Pyruvate Metabolism in Mammalian Tissues. Essays Biochem..

[53] Jeyanathan J., Martin C., Morgavi D.P. (2014). The Use of Direct-Fed Microbials for Mitigation of Ruminant Methane Emissions: A Review. Animal.

[54] Regassa A., Kiarie E., Sands J.S., Walsh M.C., Kim W.K., Nyachoti C.M. (2017). Nutritional and Metabolic Implications of Replacing Cornstarch with D-Xylose in Broiler Chickens Fed Corn and Soybean Meal-Based Diet. Poult. Sci..

[55] Huntley N.F., Patience J.F. (2018). Xylose: Absorption, Fermentation, and Post-Absorptive Metabolism in the Pig. J. Anim. Sci. Biotechnol..

[56] Turnbaugh P.J., Ridaura V.K., Faith J.J., Rey F.E., Knight R., Gordon J.I. (2009). The Effect of Diet on the Human Gut Microbiome: A Metagenomic Analysis in Humanized Gnotobiotic Mice. Sci. Transl. Med..

[57] Wang S., Wang J., Zhang J., Liu W., Jing W., Lyu B., Yu H., Zhang Z. (2023). Insoluble Dietary Fiber from Okara Combined with Intermittent Fasting Treatment Synergistically Confers Antiobesity Effects by Regulating Gut Microbiota and Its Metabolites. J. Agric. Food Chem..

[58] Simpson H.L., Campbell B.J. (2015). Review Article: Dietary Fibre-Microbiota Interactions. Aliment. Pharmacol. Ther..

[59] Stojanov S., Berlec A., Štrukelj B. (2020). The Influence of Probiotics on the Firmicutes/Bacteroidetes Ratio in the Treatment of Obesity and Inflammatory Bowel Disease. Microorganisms.

[60] Ou Y., Guo Y., Chen M., Lu X., Guo Z., Zheng B. (2023). Gut Microbiome–Serum Metabolic Profiles: Insight into the Hypoglycemic Effect of Porphyra Haitanensis Glycoprotein on Hyperglycemic Mice. Food Funct..

[61] Zhang J., Song L., Wang Y., Liu C., Zhang L., Zhu S., Liu S., Duan L. (2019). Beneficial Effect of Butyrate-Producing Lachnospiraceae on Stress-Induced Visceral Hypersensitivity in Rats. J. Gastroenterol. Hepatol..

[62] Elshaghabee F.M.F., Rokana N., Gulhane R.D., Sharma C., Panwar H. (2017). *Bacillus* as Potential Probiotics: Status, Concerns, and Future Perspectives. Front. Microbiol..

[63] Hoa T.T., Duc L.H., Isticato R., Baccigalupi L., Ricca E., Van P.H., Cutting S.M. (2001). Fate and Dissemination of Bacillus Subtilis Spores in a Murine Model. Appl. Environ. Microbiol..

[64] Casula G., Cutting S.M. (2002). Bacillus Probiotics: Spore Germination in the Gastrointestinal Tract. Appl. Environ. Microbiol..

[65] Peng J., Li X., Zheng L., Duan L., Gao Z., Hu D., Li J., Li X., Shen X., Xiao H. (2022). Ban-Lan-Gen Granule Alleviates Dextran Sulfate Sodium-Induced Chronic Relapsing Colitis in Mice via Regulating Gut Microbiota and Restoring Gut SCFA Derived-GLP-1 Production. J. Inflamm. Res..

[66] Fu R., Niu R., Li R., Yue B., Zhang X., Cao Q., Wang J., Sun Z. (2020). Fluoride-Induced Alteration in the Diversity and Composition of Bacterial Microbiota in Mice Colon. Biol. Trace Elem. Res..

[67] Zhu T., Xue Q., Liu Y., Xu Y., Xiong C., Lu J., Yang H., Zhang Q., Huang Y. (2021). Analysis of Intestinal Microflora and Metabolites From Mice With DSS-Induced IBD Treated With Schistosoma Soluble Egg Antigen. Front. Cell Dev. Biol..

