# Supplementary material for: Metabolomic Analysis of Feces vs. Cecum Content in Animals: A Comparative Study Investigated by 1H-NMR
Source: Metabolites. 2025 Aug 22;15(9):565. doi: 10.3390/metabo15090565 (PMC12471742; doi:10.3390/metabo15090565)
Supplement: Supplementary file 1 [file metabolites-15-00565-s001.zip › metabolites-3772490-supplementary.pdf]

Metabolome from Feces vs. Cecum Content in Animals: A Comparative Study Investigated by ^{1}H-NMR

XieXin Li [1], Yang Li [1], Xin Nie[1,*], Chenglin Zhu [2,*], QiQi Luo [3], Luca Laghi [4] and Gianfranco Picone [4]

Supplementary material

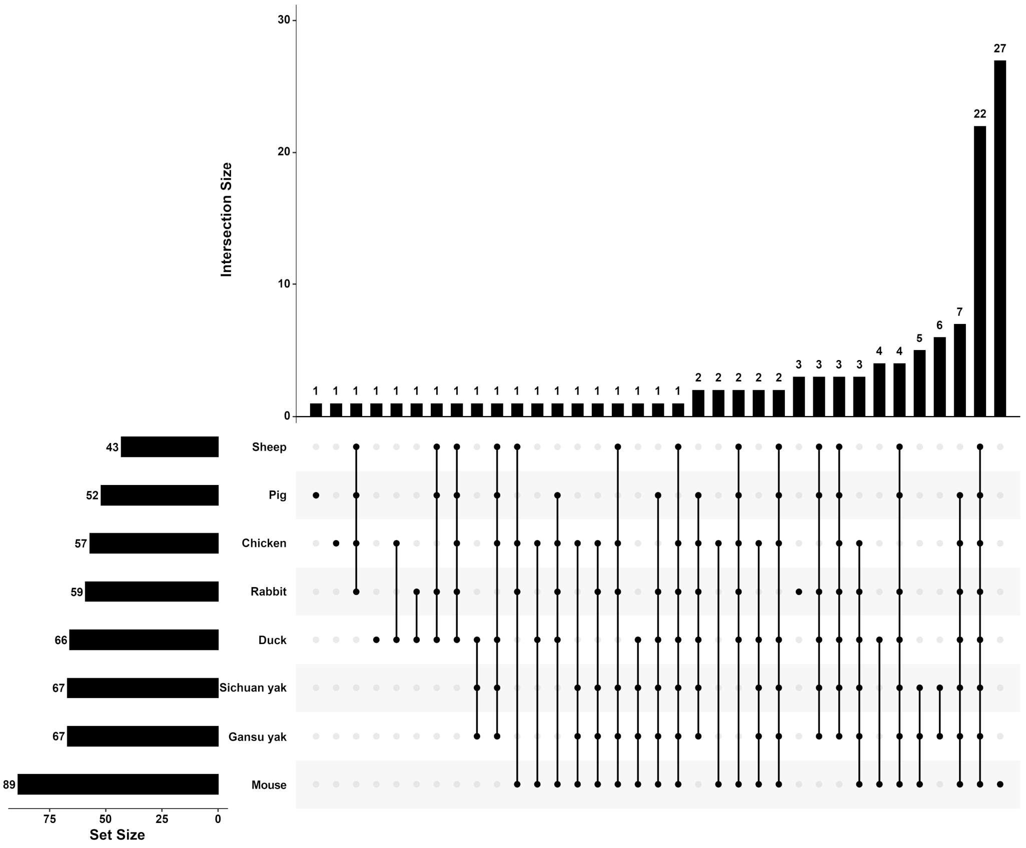

Figure S1. Upset plot showing metabolites in common and specific to the eight animals. The bar chart on the left represents the number of molecules for each animal. The line between the solid dots on the right represents the animals with common molecules. The corresponding bar chart on the top represents the number of common molecules.

Table S1. Information of molecules characterized by ^{1}H-NMR

Molecules	Functional group	Multiplicity	ppm
1,3-Dihydroxyacetone	CH_2	s	4.4111
2,3-Butanediol	CH_3	d	1.1350
2'-Deoxyinosine	CH_2	dd	3.8212
2'-Deoxyuridine	CH_2	dd	3.7607
2-Hydroxy-3-methylvalerate	CH_3-3	d	0.9373
2-Hydroxyisobutyrate	CH_3	s	1.3459
2-Hydroxyisovalerate	CH_3	d	0.8277
2-Oxoglutarate	CH_2-3	t	2.9968
2-Oxoisocaproate	CH_3	d	0.9187
3,5-Dibromotyrosine	CH_2-4	dd	3.0916
3-Hydroxybutyrate	CH_3	d	1.1931
3-Hydroxyphenylacetate	CH_2-5	s	3.4686
3-Methyl-2-oxovalerate	CH_3-3	d	1.0916
3-Phenylpropionate	CH_2-8	t	2.4808
4-Aminobutyrate	CH_2-3	t	2.2868
4-Hydroxybenzoate	CH-2	d	6.8997
4-Hydroxyphenylacetate	CH_2-4	s	3.4376
4-Pyridoxate	CH_3	s	2.4363
5-Aminopentanoate	CH_2-2	t	2.2253
Acetate	CH_3	s	1.9083
Acetoacetate	CH_3	s	2.2685
Acetoin	CH_3	s	2.2153
Acetone	CH_3	s	2.2216
Alanine	CH_3	d	1.4649
Arabinose	CH-2	dd	3.8148
Asparagine	CH_2-2	dd	2.8574
Aspartate	CH_2-2	dd	2.6765
Benzoate	CH	t	7.4738
Betaine	CH_3	s	3.2523
Bezoate	CH_2	t	7.4740
Butanone	CH_3-4	s	2.1936
Butyrate	CH_3	t	0.8820
Carnitine	CH_3	s	3.2135
Cholate	CH_3-3	s	0.9093
Choline	CH_3	s	3.1894
Creatine	CH_2	s	3.9207
Creatinine	CH_3	s	3.0334
Cytidine	CH_2	dd	3.9091

Cytosine	CH-2	d	5.9740
Dimethyl_sulfone	CH_3	s	3.1400
Dimethylamine	CH_3	s	2.7079
Ethanol	CH_3	t	1.1727
Ethylene_glycol	CH-2	tt	3.8100
Ferulate	CH_3	s	3.8991
Formate	H	s	8.4447
Fructose	CH	d	3.8832
Fucose	CH_3	d	1.2411
Fumarate	CH-2	s	5.5097
Galactose	CH-4	dd	3.6357
Gallate	CH	s	7.0346
Glucose	CH-5	dd	3.5325
Glutamate	CH-5	dd	3.7471
Glutamine	CH-5	t	3.7662
Glutarate	CH_2	t	2.1730
Glycerol	CH_2	dd	3.6394
Glycine	CH_2-2	s	3.5523
Guanidoacetate	CH_2-4	s	3.7856
Hypoxanthine	CH	s	8.1799
Isobutyrate	CH_3-2	d	1.0445
Isoleucine	CH_3	t	0.9264
Isovalerate	CH_3-2	d	0.9031
Lactate	CH_3	d	1.3240
Lactose	CH-6	t	3.2859
Leucine	CH_3-2	t	0.9493
Lysine	CH-5	t	3.7489
Malonate	CH_2	s	3.1196
Maltose	CH-3	t	3.4146
Mannose	CH-3	t	3.5668
Methanol	CH_3	s	3.3503
Methionine	CH_3	s	2.1264
Methylamine	CH_3	s	2.5974
Methylsuccinate	CH_3-3	d	1.0793
myo-Inositol	CH-2	d	3.2713
N,N-Dimethylglycine	CH_3	s	2.9144
N-Acetylglucosamine	CH_3-8	s	2.0443
Nicotinate	CH-2	d	8.9298
N-Methylhydantoin	CH_3-2	s	2.9176
N-Phenylacetylglycine	CH_2-8	d	3.7493
O-Acetylcholine	CH_3	s	2.1402
O-Phosphocholine	CH_3	s	3.2063

Ornithine	CH_2	t	3.0531
Oxypurinol	CH	s	3.2156
p-Cresol	CH_3	s	2.2466
Phenylacetate	CH_2	s	3.5269
Phenylalanine	CH_2	dd	3.1228
p-Methylhistidine	CH_3	s	3.6900
Proline	CH-3	dd	4.1186
Propionate	CH_3	t	1.0432
Propylene	CH_3	d	1.1350
Propylene_glycol	CH_3	d	1.1285
Pyridoxine	CH_3	s	2.4564
Pyruvate	CH_3	s	2.3632
Ribose	CH	dd	3.5125
Sarcosine	CH_3	s	2.7295
Serine	CH-2	dd	3.8445
Succinate	CH_2	s	2.3937
Succinylacetone	CH_3	s	2.2661
Tartrate	CH	s	4.3282
Taurine	CH_2	t	3.2514
Threonine	CH_3	d	1.3121
Thymidine	CH_2	dd	3.8301
Thymine	CH_3	d	1.8572
Trehalose	CH	t	3.4452
Trimethylamine	CH_3	s	2.8852
Trimethylamine N-oxide	CH_3	s	3.2533
Tryptophan	CH_2-5	dd	3.3038
Tyrosine	CH_2-3	dd	3.1799
Uracil	CH	d	7.5203
Uridine	CH_2	dd	3.8049
Valerate	CH_3	t	0.8763
Valine	CH_3	d	0.9826
Valproate	CH_3	t	0.8632
Xanthine	CH-2	s	7.9172
Xylose	CH	t	3.4259
